# Retraction: Application of Metabolomics and Machine Learning for the Prediction of Postmortem Interval

**DOI:** 10.7759/cureus.r156

**Published:** 2025-01-09

**Authors:** Razan Aljeaid

**Affiliations:** 1 Department of Pathology, King Abdulaziz University Faculty of Medicine, Jeddah, SAU

This article has been retracted by the Editors-in-Chief due to misrepresentation of where the work took place and the exclusion of multiple co-authors from Queen Mary University of London who contributed to the work.

